# Supplementary material for: Using Artificial Intelligence to Learn Optimal Regimen Plan for Alzheimer’s Disease
Source: medRxiv. 2023 Jan 29:2023.01.26.23285064. Preprint. [Version 1] doi: 10.1101/2023.01.26.23285064 (PMC9901063; doi:10.1101/2023.01.26.23285064)
Supplement: Supplement 1 [file media-1.docx]

**Using Artificial Intelligence to Learn Clinician's Regimen for Alzheimer's disease**

Kritib Bhattarai^a^ ,Trisha Das^b^, Yejin Kim^c^, Yongbin Chen^d^, Qiying Dai^d^, Xiaoyang Li^d^, Xiaoqian Jiang^c^, Nansu Zong^e, *^

^a^ *Department of Computer Science, Luther College Decorah, IA, United States*

^b^ *Department of Computer Science, University of Illinois Urbana-Champaign Champaign, Champaign, IL, United States*

^c^ S*chool of Biomedical Informatics, University of Texas Health Science Center, Houston, TX, United States*

^d^ *Mayo Clinic Rochester, MN, United States*

^e^ *Department of Artificial Intelligence and Informatics, Mayo Clinic Rochester, MN, United States*

^*^ *Corresponding author. Email address:* [zong.nansu@mayo.edu](mailto:zong.nansu@mayo.edu) *(Nansu Zong)*

***Supplement 1***

**
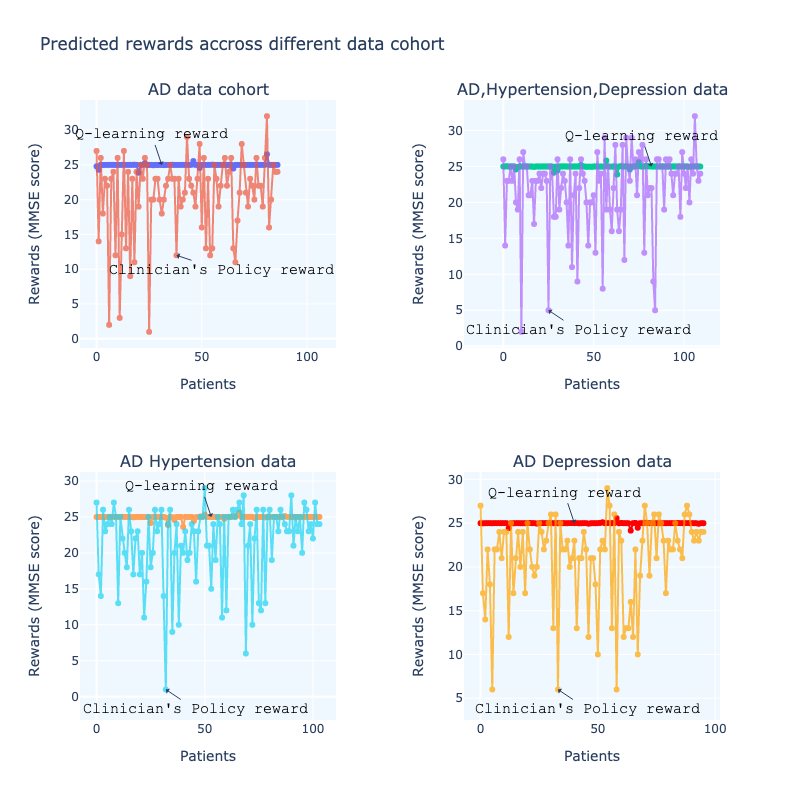
**

**Supplement Figure 1.** Rewards predicted by Q-learning and Clinician’s Policy for different Patients

***Supplement 2***

*
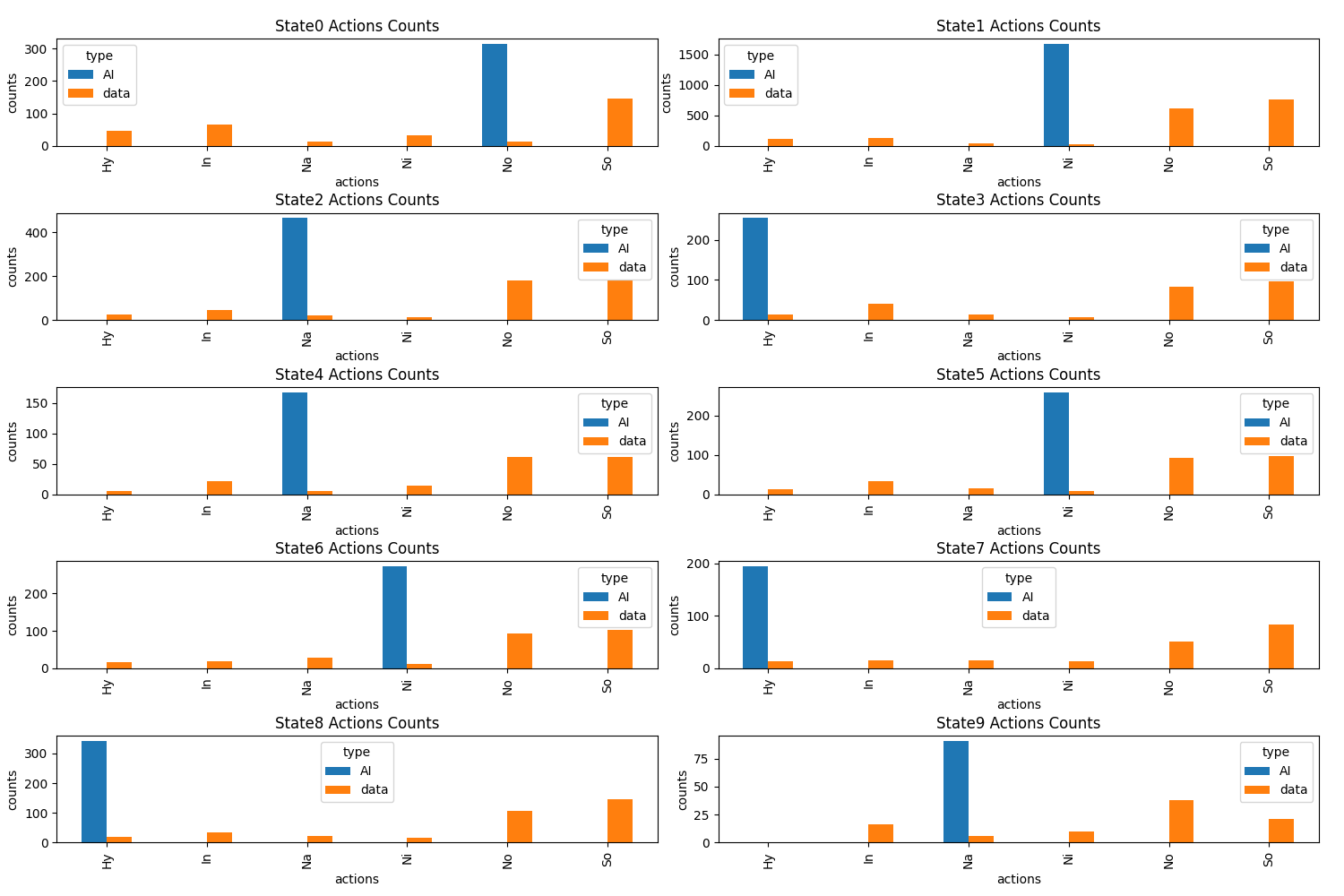
*

**Supplement Figure 2.** Drugs (actions) recommendation counts for each state by Q-learning and Clinician’s policy for AD-Hypertension-Depression Cohort. In X-axis, No is no drugs, In is inhibitors, Me is memantine, Hy is hypertension drugs, Ni is the combination of Memantine and inhibitors and So is supplements/other drugs.

***Supplement 3***


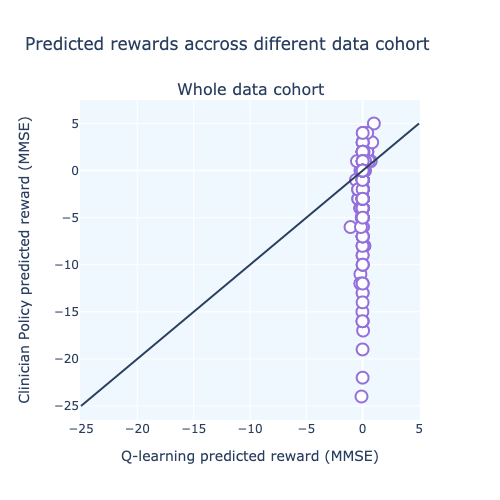


**Supplement Figure 3.** Comparison of reward prediction for different states between Q-learning and clinician’s policy for whole data

***Supplement 4***

*
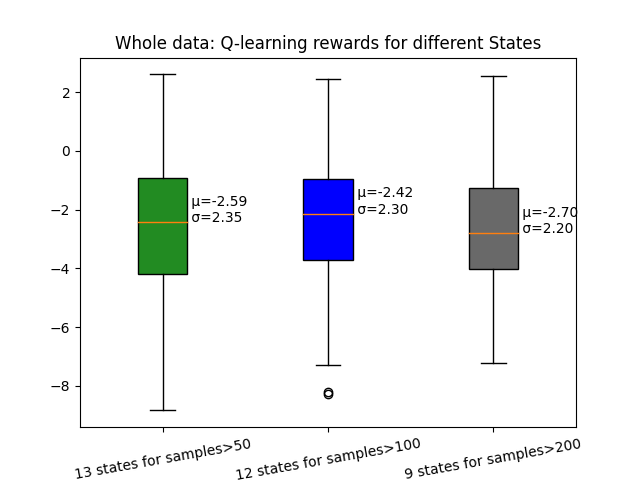
*

**Supplement Figure 4.** Comparison of Q-learning policy for the different number of states for whole data. The number of states is based on the number of samples on the leaf node of a decision tree.
